# The TreaT-Assay: A Novel Urine-Derived Donor Kidney Cell-Based Assay for Prediction of Kidney Transplantation Outcome

**DOI:** 10.1038/s41598-019-55442-x

**Published:** 2019-12-13

**Authors:** Constantin J. Thieme, Benjamin J. D. Weist, Annemarie Mueskes, Toralf Roch, Ulrik Stervbo, Kamil Rosiewicz, Patrizia Wehler, Maik Stein, Peter Nickel, Andreas Kurtz, Nils Lachmann, Mira Choi, Michael Schmueck-Henneresse, Timm H. Westhoff, Petra Reinke, Nina Babel

**Affiliations:** 1 Berlin Institute of Health Center for Regenerative Therapies (BCRT), Charité – Universitätsmedizin Berlin, corporate member of Freie Universität Berlin, Humboldt-Universität zu Berlin, and Berlin Institute of Health, Berlin, Germany; 2 Berlin-Brandenburg School for Regenerative Therapies (BSRT), Charité – Universitätsmedizin Berlin, corporate member of Freie Universität Berlin, Humboldt-Universität zu Berlin, and Berlin Institute of Health, Berlin, Germany; 3 Center for Translational Medicine, Medical Department I, Marien Hospital Herne, University Hospital of the Ruhr-University Bochum, Herne, Germany; 4 Berlin Center for Advanced Therapies (BeCAT), Charité – Universitätsmedizin Berlin, corporate member of Freie Universität Berlin, Humboldt-Universität zu Berlin, and Berlin Institute of Health, Berlin, Germany; 5 Department of Nephrology and Intensive Internal Care, Charité – Universitätsmedizin Berlin, corporate member of Freie Universität Berlin, Humboldt-Universität zu Berlin, and Berlin Institute of Health, Berlin, Germany; 6 Zentrum für Transfusionsmedizin und Zelltherapie, Charité – Universitätsmedizin Berlin, corporate member of Freie Universität Berlin, Humboldt-Universität zu Berlin, and Berlin Institute of Health, Berlin, Germany; 7 Institute for Medical Immunology, Charité – Universitätsmedizin Berlin, corporate member of Freie Universität Berlin, Humboldt-Universität zu Berlin, and Berlin Institute of Health, Berlin, Germany

**Keywords:** Predictive markers, Prognostic markers, Translational research, Kidney diseases

## Abstract

Donor-reactive immunity plays a major role in rejection after kidney transplantation, but analysis of donor-reactive T-cells is not applied routinely. However, it has been shown that this could help to identify patients at risk of acute rejection. A major obstacle is the limited quantity or quality of the required allogenic stimulator cells, including a limited availability of donor-splenocytes or an insufficient HLA-matching with HLA-bank cells. To overcome these limitations, we developed a novel assay, termed the TreaT (Transplant reactive T-cells)-assay. We cultivated renal tubular epithelial cells from the urine of kidney transplant patients and used them as stimulators for donor-reactive T-cells, which we analyzed by flow cytometry. We could demonstrate that using the TreaT-assay the quantification and characterization of alloreactive T-cells is superior to other stimulators. In a pilot study, the number of pre-transplant alloreactive T-cells negatively correlated with the post-transplant eGFR. Frequencies of pre-transplant CD161^+^ alloreactive CD4^+^ T-cells and granzyme B producing alloreactive CD8^+^ T-cells were substantially higher in patients with early acute rejection compared to patients without complications. In conclusion, we established a novel assay for the assessment of donor-reactive memory T-cells based on kidney cells with the potential to predict early acute rejection and post-transplant eGFR.

## Introduction

Kidney transplantation is the standard therapy for end-stage renal diseases. Acute (AR) or chronic rejection are among the biggest challenges in transplantation medicine, and donor reactive immunity is an important factor that counters allograft acceptance^1^. Immunosuppressive medication is given to control or prevent immune reactions; however, this medication has serious side effects. Many previous studies report heterogeneous risk profiles with respect to post-transplant complications such as AR or infections, suggesting introduction of personalized immunosuppressive therapy regimen^2–4^. For such individual therapy biomarkers that allow the discrimination between patients with different risk profiles are required^2,4–6^. The presence of donor-reactive T cells pre- and post-kidney-transplantation has been demonstrated to be associated with AR and reduced allograft survival^1,7,8^. Therefore, T cell allosensitization assays have been developed and successfully tested as predictors of the transplant outcome^7,9–12^. In these assays, the recipient’s peripheral blood cells are stimulated by alloantigen sources presenting foreign human leukocyte antigen (HLA) molecules. Currently, two sources of stimulator cells are used. One source are donor splenocytes collected during removal of the donor kidney^12,13^. However, the availability of splenocytes is limited and only feasible from deceased donors. The second source of allogenic cells is a cell bank representing the most common HLA-types^10,13^. Unfortunately, these cells often lack the adequate quality as the matching between donor and recipient HLA-types is frequently insufficient. Furthermore, since both sources are not allograft-derived, the peptides they present do not exactly resemble the tissue specific peptides presented by renal tubular epithelial cell (TEC), the main target during AR^14^. Another limitation of the currently used methods is the lack of a broad phenotypic and functional characterization of the detected alloreactive cells^3^. These underscore the need for a specific and sensitive assay for the monitoring and in-depth characterization of allograft-reactive T cells. We therefore developed the Transplant reactive T cell (TreaT)-assay by means of donor-derived renal cells obtained from recipient´s urine, a handy and renewable antigenic source for stimulation. Using multiparameter flow cytometry, we showed that our assay has the potential to predict the graft function post-transplantation and early episodes of AR and could thus serve as an important tool for guiding precision immunosuppressive regimens in kidney transplant patients. Furthermore, the characterization of alloreactive T cells has the potential to reveal new molecules involved in the process of AR.

## Materials and Methods

**Figure 1:**
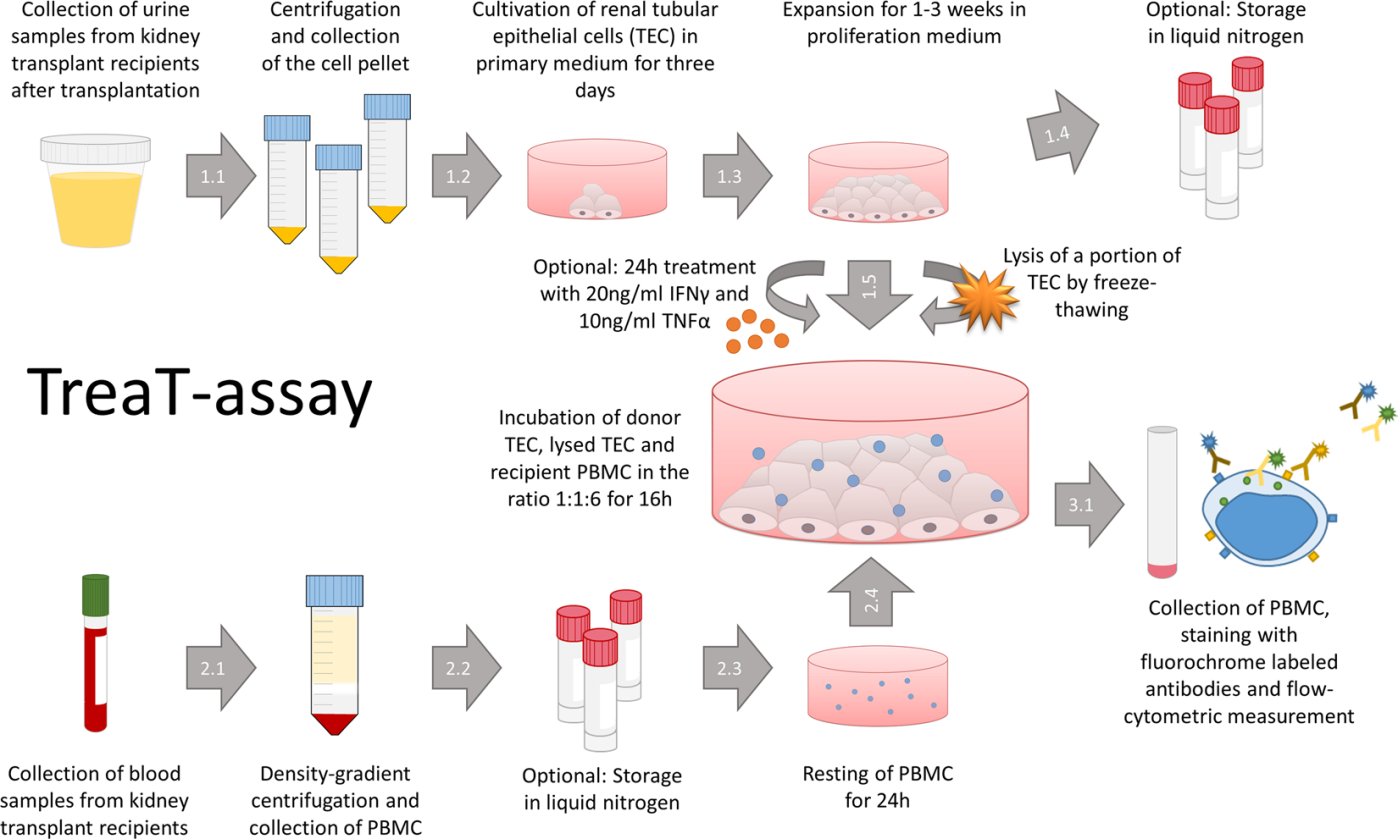
Methodological overview of the TreaT-assay. For a detailed description please consult the materials and methods section of the main text.

An overview of the TreaT-assay setup can be found in Fig. 1.

### Patients

Four healthy volunteers as well as in total twenty-two patients transplanted in the Charité - Universitätsmedizin Berlin were recruited and analyzed in a cross-sectional study for the assay establishment at different time-points pre- and post-transplantation. No organs were procured from prisoners. The number of patients and samples analysed in the study differed in dependence from the aim addressed in the study. Thus, 22 patients were recruited for the cultivation procedure of TEC. For analysis of HLA expression on TEC, samples of 18 patients were used. For the assay establishment, samples of 13 patients were collected. The prediction of eGFR were performed using the samples of 14 patients, and 12 patients were followed up clinically for the prediction of acute rejection. For the comparison of stimulatory capacity between splenocytes and TEC, samples of 4 patients were collected (Supplementary Table S1). All patients gave written informed consent and the study was approved by the ethics commission of the Charité – Universitätsmedizin Berlin, Germany in accordance with the declaration of Helsinki.

### Collection of peripheral blood mononuclear cells (PBMCs)

Blood was collected from each kidney transplant recipient recruited into the study at least once and up to four times in Vacutainers containing lithium heparin (Beckton Dickinson (BD), Franklin Lakes, US-NJ). The PBMCs were isolated by density gradient centrifugation Ficoll-Paque (Biochrom, Berlin, Germany). The cells were frozen in CryoPure tubes (Sarstedt, Nümbrecht, Germany) in Roswell Parm Memorial Institute-1640 medium (RPMI, Biochrom) with 60% fetal bovine serum (FBS, Biochrom) and 10% dimethylsulfoxid (DMSO; Sigma-Aldrich, Munich, Germany) at −80 °C in a freezing box (ThermoFisher, Waltham, US-MA) containing isopropylalcohole (Sigma-Aldrich) and stored in liquid nitrogen until further usage. Upon defrosting, the cells were left resting for 24 hours.

### Cultivation of tubular epithelial cells

Up to 300 ml of urine were collected in sterile flasks (Corning Falcon, Corning, US-NY) within the first week after kidney transplantation. To ensure the donor origin of urine cells, urine was collected from mono pigtail stents in patients with existing residual diuresis. The samples were then processed further according to a previously published protocol^15^. Briefly, the urine sediment was washed with phosphate buffered saline (PBS, ThermoFisher) and seeded in primary medium, containing Dulbecco’s modified Eagle medium (DMEM, ThermoFisher) and Ham’s F12 (Biochrom) in a 1/1 ratio, 10% FBS, renal epithelial growth medium (REGM) SingleQuot kit (Lonza Clonetics), 100 U/ml penicillin/streptomycin (P/S, Biochrom), 2.5 µg/ml amphotericin B (Biochrom), 100 µg/ml normocin (InvivoGene) and 10 µg/ml ciprofloxacin (Fresenius Kabi Austria). After three days, the medium was replaced by the proliferation medium, containing renal epithelial basal medium (REBM, Lonza, Basel, Swiss), REGM SingleQuot kit, 10% FBS, 2.5 mM GlutaMAX (ThermoFisher), 1% non-essential amino acids (ThermoFisher) and 100 U/ml / 100 µg/ml P/S. Mycoplasma contamination was monitored in random samples (MycoAlert, Lonza). The cells were cultivated until confluency, harvested using trypsin/EDTA-solution (Biochrom), and frozen as described above until further usage.

### Induction of HLA expression

Frozen tubular epithelial cells (TEC) were thawed, seeded in two cell-culture flasks of 75 cm² (Coning Falcon), and cultivated in proliferation medium until confluency. Then, the flasks were incubated with RPMI containing 10% FBS and 100 U/ml P/S with or without supplementation of 20 ng/ml interferon γ (IFNγ) and 10 ng/ml tumor necrosis factor α (TNFα) (both Miltenyi Biotec, Bergisch-Gladbach, Germany) to enhance or induce surface HLA-ABC and -DR expression. After 24 h, the TEC were harvested. A fraction of the cells was analysed by flow cytometry and the rest was used as stimulators in an alloantigen-assay.

### Cell lysis

To mimic tissue damage and to facilitate presentation of alloantigens by antigen presenting cells, a fraction of TEC were lysed by centrifugation with 4500 g, vortexing and repeated freeze-thawing at −20 °C in RPMI until further usage.

### Preparation of donor splenocytes

Pieces of at least 6 cm of spleen from kidney-transplant donors were used for further preparation. Splenic connective tissue was removed with sterile forceps and scalpel (Feather, Osaka, Japan) and minced through a 100 µm and a 40 µm sterile cell strainer sieve (BD) with PBS. Mononuclear cells were isolated by Ficoll gradient and frozen as described above. Frozen splenocytes were thawed in RPMI containing 1% DNAse (Roche Diagnostics, Rotkreuz, Swiss) 12 h prior to use in the alloantigen-assay. A fraction of the splenocytes was lysed as described above. To discriminate recipient’s PBMC and donor splenocytes in flow cytometry, the remaining splenocytes were labelled with 5 µM carboxyfluorescein succinimidyl ester (CFSE, Sigma) one hour before cultivation in the alloantigen-assay.

### Alloantigen-assay

0.25×10^6^ TEC treated with or without IFNγ- and TNFα were seeded into 24-well-plates (Corning Falcon). 1.5×10^6^ recipient PBMCs and lysates of 0.25×10^6^ TEC were added (ratio TEC:lysed TEC:PBMC = 1:1:6). For the experiments comparing autologous with allogenic stimulation, TEC isolated from two healthy volunteers were incubated with fresh, not cryopreserved PBMCs of the same or a different donor. PBMCs seeded into wells without TEC and lysed TEC served as the negative control. The positive control was treated with phorbol 12-myristate 13-acetate (PMA, Sarstedt) and ionomycin (Iono, ThermoFisher). All wells were incubated with 1 µg/ml brefeldin A (Sigma) and 1 µl/ml protein transport inhibitor containing monensin (BD). Additional specimen with 0.25×10^6^ CFSE labelled kidney transplant donor-derived splenocytes and 0.25×10^6^ lysed splenocytes as stimulators for 1.5×10^6^ recipients’ PBMCs were set. After 16 hours of incubation the PBMCs were harvested and stained for flow cytometry.

### Flow cytometry staining of TEC and PBMCs

Cell surface of PBMCs and splenocytes was stained with anti-CD161 Brilliant Violet (BV) 510 (clone HP-3G10, Biolegend, San Diego, US-CA) and Live/Dead Blue (ThermoFisher) to exclude dead cells. Intracellular staining of PBMCs was performed with FoxP3-Permeabilization Buffer (ThermoFisher) and with anti-CD3 Brilliant Ultraviolet 737 (clone UCHT1, BD), anti-CD4 BV650 (clone OKT4, Biolegend), anti-CD8 allophycocyanin (APC)/Cy7 (clone RPA-T8, Biolegend), anti-CD137 phycoerythrin (PE)/Cy5 (clone 4B4-1, BD), anti-CD154 peridinin-chlorophyll-protein (PerCP)/Cy5.5 (clone 24-31, Biolegend) and anti-granzyme B Alexa Fluor 700 (clone GB11, BD).

Cell surface of TEC was stained with Live/Dead Blue, anti-CD13 APC (clone WM15, Biolegend), anti-CD90 PerCP/Cy5.5 (clone 5E10, Biolegend), anti-CD326 (EpCam) FITC (9C4, Biolegend), anti-HLA-ABC PE (clone 311506, Biolegend) and anti-HLA-DR APC/Cy7 (clone L243, Biolegend). Anti-cytokeratin BV421 (clone CAM5.2, BD) was administered for intracellular staining. Anti-HLA-ABC PE and anti-HLA-DR APC/Cy7 were also used for staining of splenocytes.

Patient samples were measured using LSRFortessa (BD) while healthy donors were measured using CytoFlex LX (Beckman Coulter, Brea, US-CA). Gating and analysis was performed with FlowJo version 10 (FlowJo LLC, Ashland, US-OR). Analyses of patient samples and healthy blood donors by two different flow cytometers were not critical, since the data of both populations were not directly compared to each other in the study.

### Statistical analysis and graphical representation

Gating of flow cytometry data was performed with unstained, untreated, and adequate fluorescence minus one (FMO) controls according to standard flow cytometry guidelines^16^. HLA expression on TEC was analysed as median fluorescence intensity (MFI). CD137/CD154^+^ CD4^+^ and CD137^+^ CD8^+^ T cells and their subsets were analysed as cells per 10^6^ CD4^+^ and CD8^+^ T cells, respectively. Counts of less than two cells per gate were excluded. To account for specifically activated cells and their phenotype, the corresponding population in the negative control was subtracted, except for the experiments to show the specificity of the assay where the negative control is depicted for a better illustration of the background T cell activity. Statistical analysis was performed with Prism 7 (GraphPad Software, San Diego, US-CA). Gaussian distribution was assessed using D’Agostino & Pearson normality test and Shapiro-Wilk normality test. Parametric or non-parametric statistical tests were used accordingly as indicated. In detail, comparison of HLA expression as well as the reactivity of recipient PBMC to donor UC and splenocytes was done with Wilcoxon matched-pairs signed rank test for not normally distributed samples. To test the ability of UC to stimulate alloantigen-reactive T cells, non-parametric statistics was done with Friedman-Test and Dunn’s multiple comparison test. As sphericity was not assumed, the Greisser-Greenhouse correction was applied. Correlation of eGFR (calculated with CKD-EPI) and pre-transplant alloreactive T cells was calculated with Pearson’s correlation coefficient.

## Results

### Generation of urine-derived tubular epithelial cells and induction of HLA-ABC and –DR expression

**Figure 2:**
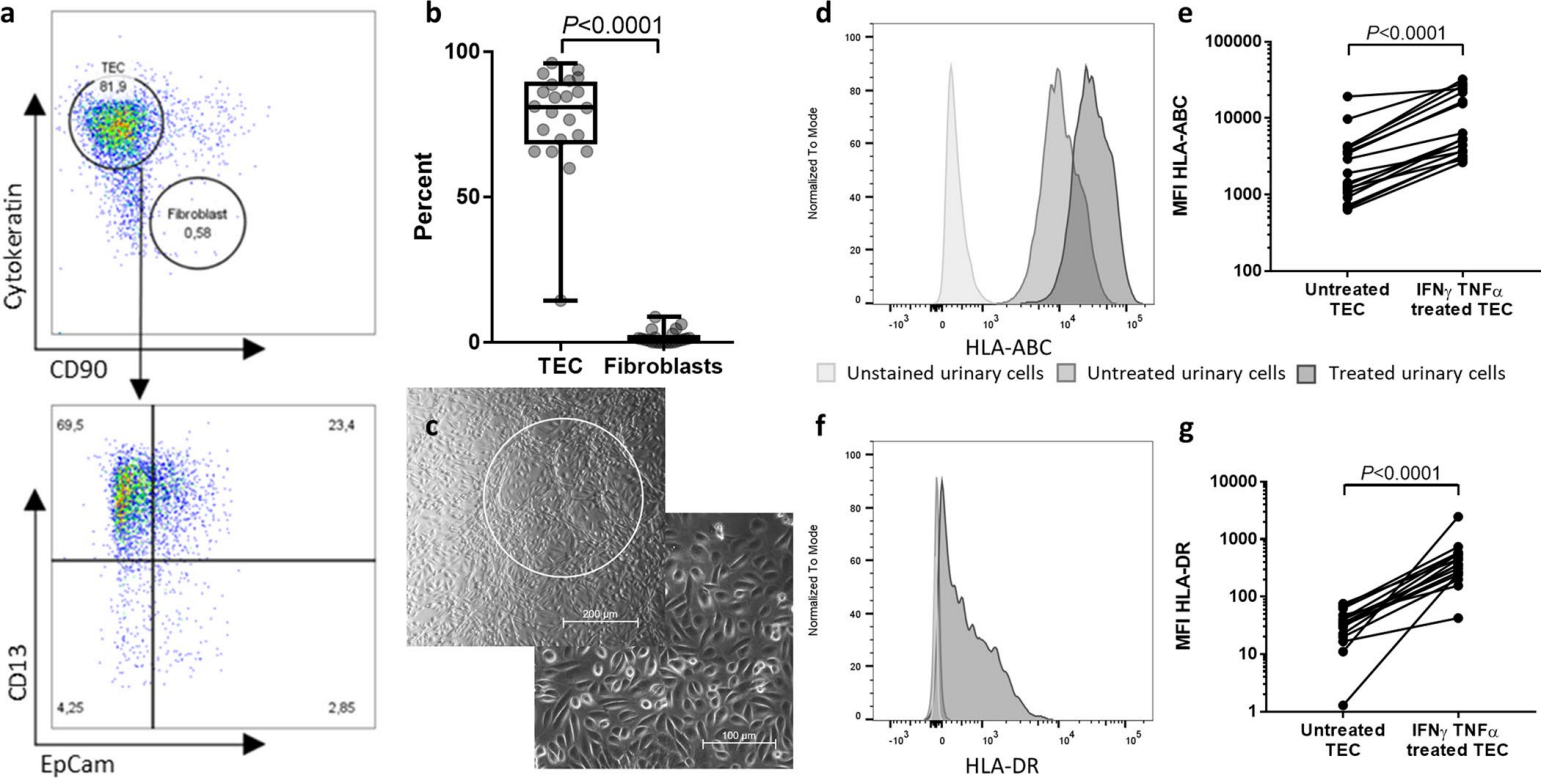
Urinary culture cells mainly consist of tubular epithelial cells (TEC) and present HLA-ABC and -DR-molecules upon IFNγ and TNFα treatment. (**a**) Representative flow cytometric characterization of urinary culture cells. Urine was collected from kidney-transplant patients after transplantation and the cell pellet was seeded in culture media after centrifugation. The colonies were then expanded in proliferation media for 1–3 weeks. After harvesting, the cells were stained for epithelial cell marker cytokeratin, proximal and distal renal tubular cell markers CD13 and EpCam, and for fibroblast marker CD90. One representative example of 22 individual donors is demonstrated. (**b**) Quantification of TEC (cytokeratin^+^ CD90^-^) and Fibroblasts (cytokeratin^-^ CD90^+^ ) among living urinary culture cells (*n* = 22). Cells were analysed after 3–6 weeks in culture. Statistical comparison was done with Wilcoxon matched-pairs signed rank test for not normally distributed samples. (**c**) Representative phase-contrast microscopy of two samples of urinary cell cultures. The upper picture shows the characteristic dome formation of TEC (white circle). The cell morphology shown in the lower picture indicates epithelial cells. Magnification 20 × (left) and 10 × (right). A representative example for 22 individual donors is shown. (**d**–**g**) Expression levels indicated by median fluorescence intensity (MFI) of HLA-ABC (**d**,**e**) and HLA-DR ((**f**,**g**) on urine-derived TEC with and without addition of 20 ng/ml IFNγ and 10 ng/ml TNFα for 24 h (*n* = 18). Harvested cells were analysed by flow cytometry. Histograms are representative for n = 18. Statistical analysis was done with Wilcoxon matched-pairs signed rank test for not normally distributed samples.

In total, four healthy individuals and twenty-two kidney-transplant patients were included in a cross-sectional study in order to establish our novel methodological approach. Cells were cultured from all twenty-two patients and two healthy individuals. Flow cytometric expression of cytokeratin, CD13, and epithelial cell adhesion molecule (EpCam), which define tubular epithelial cells (TEC)^17,18^, as well as CD90 as a marker for contaminating fibroblasts^19^ was analysed. We found that the vast majority of the cultivated cells consists of TEC (median 81%, IQR 69–89%), mainly proximal (CD13^+^) and to a lesser extent distal (EpCam^+^) TEC (Fig. 2A,B). Microscopical evaluation confirmed the epithelial phenotype and the dome formation that is characteristic for TEC (Fig. 2C). We will therefore refer to the cells cultured from the urine as TEC in the following paragraphs.

To mimic the inflammatory environment during rejection and enhance HLA expression, the TEC were treated with IFNγ and TNFα for 24h^20^. The flow cytometric analysis of the HLA expression confirmed an increase of HLA-ABC on TEC when compared to the untreated sample (Fig. 2D,E; *P* < 0.0001, *n* = 20). Similarly, HLA-DR on TEC significantly increased after TNFα and IFNγ treatment (Fig. 2F,G; *P* < 0.0001, *n* = 20). Taken together, as it has been shown before for TEC derived directly from donor kidneys^21^, our data portray the ability of urine-derived TEC to act as atypical antigen-presenting cells.

### Alloreactive T cells can be stimulated with urine-derived TEC

To examine whether we can induce a specific activation of alloantigen-reactive T cells, we analysed the activation marker profile of T cells following 16 h of co-cultivation of recipient PBMC with respective donor TEC. Lysed TEC were added to intact TEC to facilitate presentation by antigen presenting cells and to mimic ischemic cell death that can occur during transplantation. Recipient PBMC alone were used to define the T cell baseline activation. For the assay establishment, PBMCs from 13 patients were obtained, some of them at different time points, so 30 samples were analysed in total.

**Figure 3:**
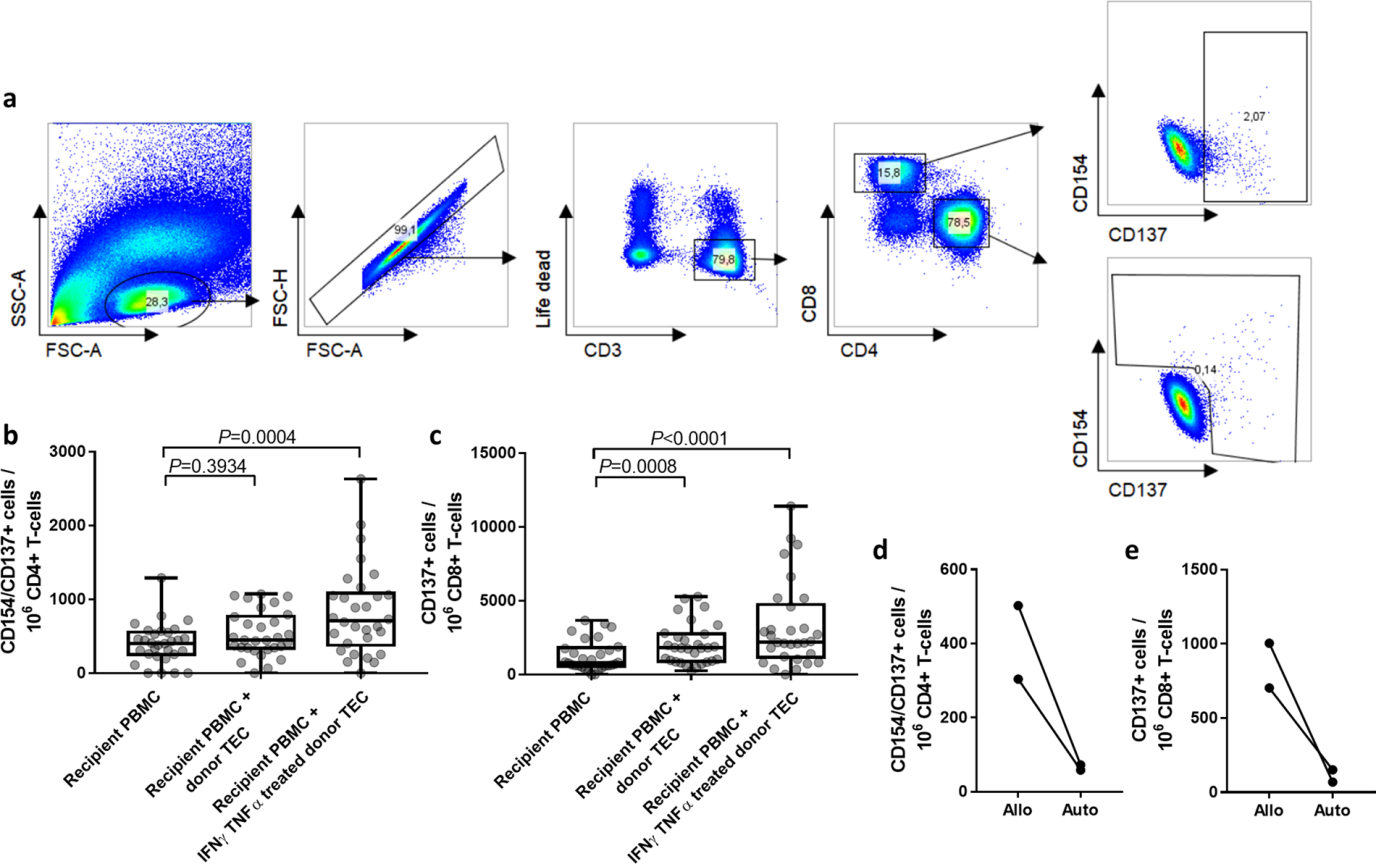
Alloreactive T cells can be monitored with urine-derived donor TEC. **(a**) Representative flow cytometry plots illustrating the gating strategy to identify single living alloreactive CD3^+^ CD4^+^ CD154^+^/CD137^+^ and CD8^+^ CD137^+^ lymphocytes. From left to right: Scatter plot of PBMCs distinguishing lymphocytes from debris and non-lymphocytes; doublet exclusion; discrimination of live CD3^+^ T cell from dead and non-T cells; discrimination of CD4^+^ and CD8^+^ T cells; identification of reactive T cells according to CD137 and CD154 expression. (**b**–**c**) Urine-derived donor TEC can elicit a donor-reactive activation of recipients’ CD4^+^ (**b**) and CD8^+^ (**c**) T cells. PBMC of 13 recipients obtained at different time points (*n* = 30) were co-cultivated for 16 h with lysed and intact urine-derived donor TEC or with no further stimuli (negative control). The specific stimulation was further compared between donor TEC treated with 20 ng/ml IFNγ and 10 ng/ml TNFα for 24 h or untreated TEC. Activation of T cells was assessed by flow cytometric determination of CD4^+^ CD137^+^/CD154^+^ and CD8^+^ CD137^+^ T cells as described in (**a**). Statistical comparison between the three experimental groups was performed with Friedman test and Dunn’s multiple comparisons test. (**d**–**e**) Urine-derived TEC do not elicit an activation of autologous CD4^+^ (**d**) and CD8^+^ (**e**) T cells. TEC of two healthy donors were cultivated, treated with 20 ng/ml IFNγ and 10 ng/ml TNFα, partially lysed and incubated with autologous (auto) or randomly selected allogenic (allo) PBMCs (*n* = 4 PBMC donors). T cell activation was assessed as described above.

Activated CD4^+^ T cells were defined as activation marker CD154 and CD137 positive cells (Fig. 3A). Compared to the untreated controls we observed a significantly higher number of activated CD4^+^ T cells in cultures where the TEC have been stimulated with IFNγ and TNFα (Fig. 3B; *P* = 0.0004, *n* = 30). Of note, untreated TEC did not induce specific activation of CD4^+^ T cells (*P* = 0.3934, *n* = 30).

With regard to CD8^+^ T cells, we observed an even higher proportion of alloreactive cells compared to the CD4^+^ T cells. Cytokine-treated TEC induced a significantly higher number of CD137^+^ CD8^+^ T cells compared to the negative control (Fig. 3C; *P* < 0.0001, *n* = 30). In contrast to CD4^+^ T cells, they could also be activated by untreated TEC (*P* = 0.0008, *n* = 30), although the magnitude of the response was lower.

To exclude that the observed reactivity reflects an unspecific reaction to cultivated and lysed TEC, we performed additional experiments with randomly selected healthy individuals (*n* = 4), where we used cytokine-treated TEC of two of these individuals as stimulators for either PBMCs of the same donor (auto) or of an allogenic donor (allo) (Fig. 3D,E). The healthy volunteers were randomly selected without specific HLA recruitment criteria as previously published^22^. While the frequencies of autologous activated CD4^+^ and CD8^+^ T cells were negligible, allo-specific CD4^+^ and CD8^+^ T cells were detectable (304–502.7 for CD4^+^ and 703.5–1004.2 for CD8^+^ alloreactive T cells/10^6^ T cells). Collectively, these data show that donor-reactive T cells can be detected using the assay based on urine-derived TEC of kidney transplant patients.

### Donor-TEC demonstrate superior stimulatory capacity compared to donor-splenocytes

**Figure 4:**
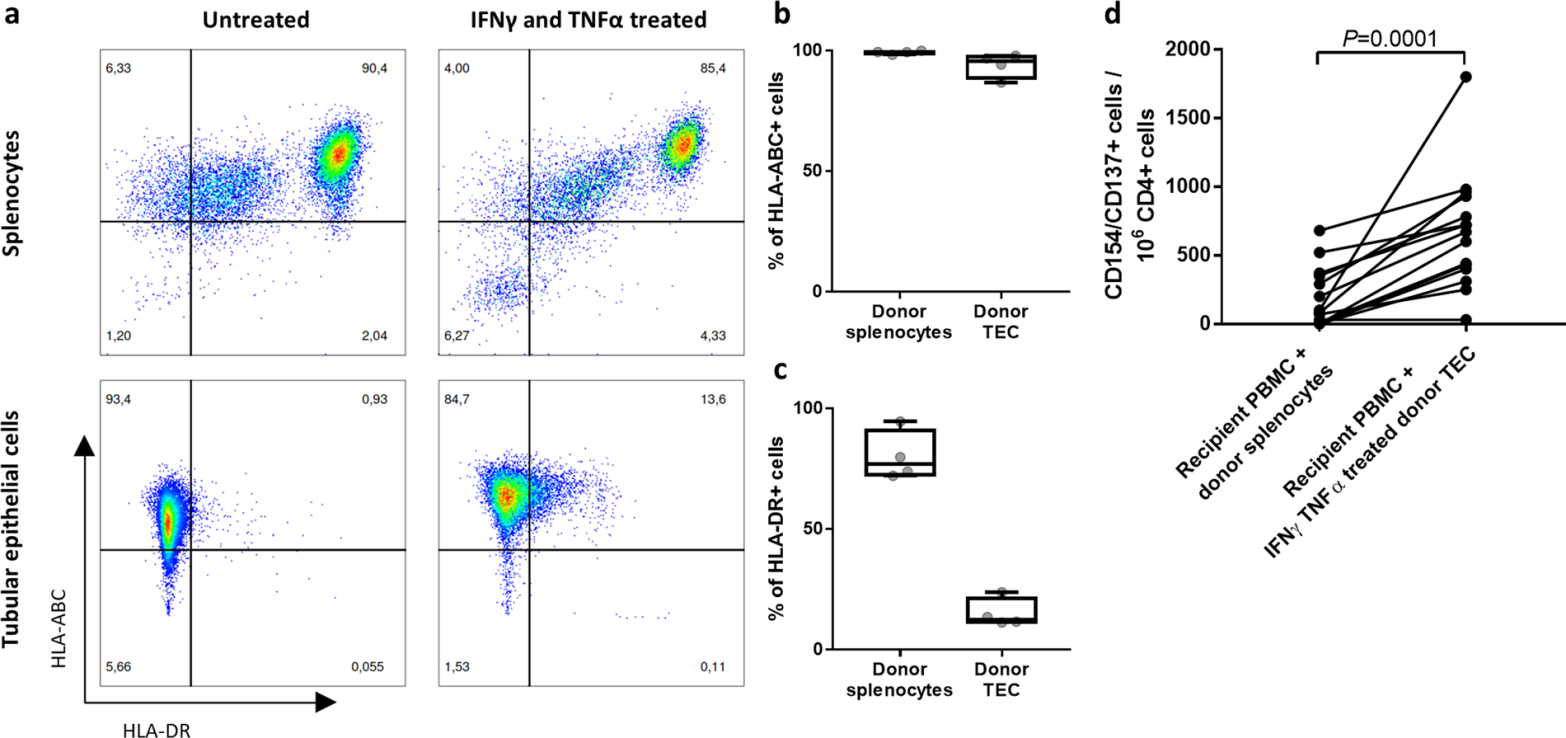
Assessment of alloreactive T cells using urine-derived donor TEC-stimulation shows higher sensitivity compared to donor splenocytes-stimulation. (**a**) Splenocytes express higher levels of HLA-DR than TEC. Representative plots demonstrating flow cytometric analysis of HLA-ABC and HLA-DR expression on untreated and 24 h IFNγ and TNFα treated urine-derived TEC or splenocytes. Both cell types were derived from the same deceased donor. (**b**,**c**) Frequencies of HLA-ABC (**b**) and HLA-DR (**c**) expressing living splenocytes and treated living TEC determined by flow cytometry (n = 4). (**d**) TEC elicit a higher alloreactivity than splenocytes from the same donor. PBMCs of four kidney-allograft recipients of different time points (n = 15) were stimulated with IFNγ and TNFα treated TEC and lysed TEC or splenocytes and lysed splenocytes, both derived from the kidney-transplant donor. Specific activation was assessed by flow cytometric measurements of the activation marker CD154 and CD137 expression on CD4^+^ T cells. Statistical analysis was done with Wilcoxon matched-pairs signed rank test.

Usage of donor-splenocytes for monitoring alloreactive T cells is a well-established and elegant method, but only applicable for deceased donations. Urine-derived TEC offer the advantages of an unlimited availability of cells directly from the transplanted organ. We performed our assay in four kidney-transplant patients with both stimulator cell types from the corresponding donor to compare the amount of detectable alloantigen-reactive T cells. Analysis of the HLA-ABC and -DR expression on splenocytes showed a high expression of both molecules. After treatment with IFNγ and TNFα the expression slightly decreased (Fig. 4A, upper row), which might be due to a decreased viability (not shown). In comparison, the basal expression of HLA-DR on the TEC of the same donor was very low and inducible by IFNγ and TNFα treatment, but stayed lower in comparison to splenocytes (Fig. 4A–C). To compare the stimulatory capacity of the TEC, as a novel source of stimulator cells directly from the kidney allograft, to splenocytes from the same donor as the most commonly used source, we analysed the respective allograft-reactive T cell responses. We lysed a fraction of the TEC and splenocytes and incubated them together with the respective intact cells to facilitate presentation by antigen presenting cells and to mimic ischemic cell death that can occur during transplantation. The analysis was performed in four patients at different time points pre- and post-transplantation, making a total of 15 samples. We detected on average 8 times higher median frequencies of activated CD4^+^ T cells in samples stimulated with donor TEC compared to donor-derived splenocytes (Fig. 4D; *P* = 0.0001, *n* = 15). These data thus show that TEC can elicit a stronger donor-specific CD4^+^ T cell response than splenic antigen presenting cells can.

### Characteristics of patients in follow up-study

It has been shown before that measurement of pre-transplant donor-reactive T cells can predict the post-transplantation kidney-function and AR^13,23–26^. To confirm the capacity of our method to quantify donor-reactive T cells and predict the post-transplant clinical course we collected PBMCs of fourteen patients before transplantation. Thereafter, the patients were clinically followed up for 6 months after transplantation. Two patients developed biopsy-proven early acute rejection (AR group). Ten patients showed a stable graft function and no signs for delayed graft function, AR or other relevant post-transplant complications (control group). One patient showed multiple surgical and infectious complications post-transplantation. Another patient showed a good transplant function initially but a deterioration of kidney function two weeks after transplantation. Both patients had single transplant-biopsies with inconclusive findings regarding immunological complications, so they were not included into the analysis. The demographic and clinical characteristics of the 10 control patients and the 2 AR patients are presented in Table 1. Briefly, eight control patients were male and two female. Their age at transplantation was 64.5 (32–77) years (median, range). The immunosuppression regimen of nine control patients consisted of tacrolimus, mycophenolat-mofetil and methylprednisolone, one patient received a regimen with everolimus, cyclosporine and methylprednisolone. Two of the twelve monitored patients developed a biopsy-proven cellular AR at early stage after transplantation, confirmed via biopsy. They were both male and their median age at transplantation 73.5 (74–76) years (median, range). Their therapeutic regimen after transplantation consisted of a maintenance immunosuppression with tacrolimus, mycophenolate-mofetil, and methylprednisolone.

**Table 1:** Characteristics of control and acute rejection (AR) patients in follow-up TreaT-assay.

Parameter		Control patients (No DGF, no AR) *n* = 10	AR-patients *n* = 2
Recipient	Age *year, median (range)*	64.5 (31–77)	73.5 (73–74)
	Sex *male/female*	8/2	2/0
	Underlying renal disease	Glomerulonnephritis (*n* = 3), diabetic nephropathy (*n* = 1), hypertensive nephropathy (*n* = 1), chronic pyelonephritis (*n* = 1), interstitial nephritis (*n* = 1), CNI-toxicity after previous lung-tx (*n* = 1) and unknown (*n* = 2)	Autosomal dominant polycistic kidney disease (*n* = 1), glomerulonephritis (*n* = 1)
	Previous kidney transplant	10%	50%
	Previous transplant other than kidney	10%	0%
	Time on dialysis before Tx *years, median (range)*	5 (3–11)	12 (12)
	Current PRA	0%	0%
	Induction immunosuppression	Basiliximab	Basiliximab
	Maintenance immunosuppression	Tacrolimus, mycophenolat-mofetil, methylprednisolone (*n* = 9) Cyclosporine, everolimus, methylprednisolone (*n* = 1)	Tacrolimus, mycophenolate-mofetil, methylprednisolone
Donor-recipient	HLA-mismatches broad *median (range)*	4 (0–6)	5 (4–6)
	Cold ischemia time *hours, median (range)*	7 (4–17)	12 (9–15)
Donor	Age *year, median (range)*	59 (23–74)	75 (74–76)
	Sex *male/female*	4/6	1/1

### Pre-transplant alloreactive T cells measured with the TreaT-assay correspond with the post-transplant outcome

**Figure 5:**
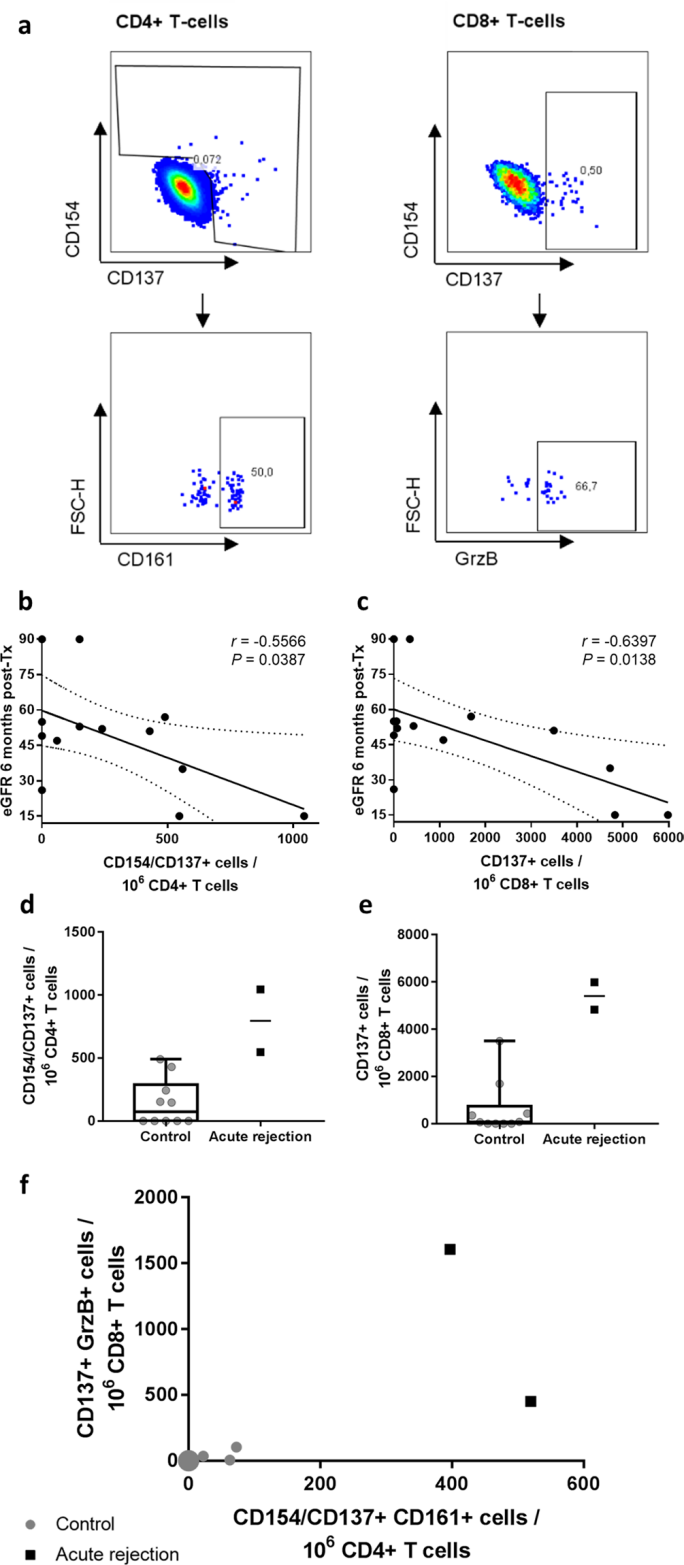
Pre-transplant alloreactive T cells measured with the TreaT-assay correspond with the post-transplant outcome. (**a**) Representative gating strategy to identify allograft-reactive T cell subsets. (**b**,**c**) Analysis of donor-reactive T cells collected before transplantation (*n* = 14 patients). Recipient’s PBMC were stimulated with the corresponding urine-derived donor TEC for 16 h. Before incubation, the TEC were treated with 20 ng/ml IFNγ and 10 ng/ml TNFα for 24 h and a fraction of the TEC lysed to facilitate presentation by antigen presenting cells. After incubation, the PBMC were analyzed by flow cytometry for frequencies of alloreactive T cells. Correlation with the eGFR (CKD-EPI) at 6 months post-transplantation with pre-transplant alloreactive CD4^+^ (**b**) and CD8^+^ (**c**) T cells was calculated with Pearson’s correlation coefficient. Dotted lines show 95% confidence bands. (**d**–**e**) Two patients of the previously described cohort developed biopsy-proven early acute rejection (AR) in clinical follow-up, and the frequencies of their pre-transplant alloreactive CD4^+^ (**d**) and CD8^+^ (**e**) T cells were compared to ten patients without immunological complications in the first six months after transplantation (control group). Whiskers show minimal and maximal values. (**f**) Further characterization of alloreactive CD4^+^ T cells by the expression of TH17 marker CD161 and of alloreactive CD8^+^ T cells by production of Granzyme B. Gray dots show control patients (*n* = 10), the large gray dot conjoins 7 individual patient data. Black squares show patients with biopsy-proven early AR (*n* = 2).

To determine phenotypical and functional subsets of alloreactive T cells we applied multi-color flow cytometry (Fig. 5A). The pre-transplant numbers of alloreactive T cells correlated negatively with the eGFR (CKD-EPI) 6 months post-transplantation. In detail, this could be seen for alloreactive CD4^+^ (*r* = −0.5566, *P* = 0.0387, *n* = 14) as well as CD8^+^ (*r* = −0.6397, *P* = 0.0138, *n* = 14) T cells (Fig. 5B,C).

To confirm that the established assay can be used to predict early AR episodes, twelve transplant patients were grouped into a control group without immunological complications (*n* = 10) and an AR group with biopsy proven rejection (*n* = 2) and analysed in a pilot study. The frequencies of alloreactive T cells were measured in samples obtained immediately before transplantation. The number of alloreactive CD4^+^ and CD8^+^ T cells before transplantation was higher in the two AR patients compared to controls. Comparing these results by a Mann-Whitney test resulted in significant differences (*P* = 0.0152 in both subsets), but as applying statistical tests in experiments with low sample numbers is controversial we refrained from depicting the *P*-value (Fig. 5B,C). The number of allograft-reactive CD154^+^ and/or CD137^+^ CD161^+^ CD4^+^ T cells and of CD137^+^ granzyme B producing CD8^+^ T cells could clearly distinguish between patients that developed an early AR and patients with an uncomplicated course after transplantation (Fig. 5D). CD161 is a marker for TH17 cells^27^, which are important players in the T cell reaction towards the allograft^28^. Granzyme B has been introduced as a marker for AR after kidney transplantation before^29^.

## Discussion

T cell-mediated rejection is a common threat after kidney transplantation^1^. Donor-reactive T cells suggested to be involved in pathogenesis of AR can be generated prior to transplantation, for example during pregnancies, blood transfusions, previous transplantations or as cross-reactive T cells during infections^30^. They are of special interest, since they may allow the prediction of the immediate post-transplant clinical course^8^. CD4^+^ and CD8^+^ memory T cells can act without secondary lymphoid organs^31^, which enables monitoring their reactivity *ex vivo* in a short-term stimulation approach^32^. Previously, we and others could show that the number of pre-transplant donor-reactive IFNγ-producing cells measured by ELISPOT correlates with post-transplant glomerular filtration rate^23–25^ and predicts early AR^13,26,33,34^. However, stimulator cells applied in these assays pose several limitations. They are either of restricted availability (splenocytes) or lack sufficient matching (HLA-bank cells). Furthermore, functional and phenotypic analysis of alloreactive cells with ELISPOT applied in previous studies is very limited^3^. Here, we present the Transplant reactive T cells (TreaT)-Assay, a novel multi-parameter flow cytometry-based diagnostic tool using an easily accessible and renewable urine-derived donor-specific source of stimulator cells for monitoring allograft-specific T cells. Compared to the currently used sources our model has the advantages of high quantity, availability, and quality.

The cultivation process is easy to perform. The outgrowth of cells from the urine worked for all patients included in our study. As we could show, the majority of cells in the cultures are TEC. Since the urine was collected from a pigtail catheter, the allograft origin of TEC can be ensured. Therefore, this method offers a non-invasive way to procure kidney allograft cells.

Regarding the quality, urinary cells have been shown earlier to be fully functional renal tubular cells^35^. Most important in our setting is their stimulatory capacity. We could show that urine-derived TEC, like TEC from other sources^21,36^, up-regulate both HLA-ABC and –DR molecules in a pro-inflammatory environment. These cells can therefore act as atypical antigen presenting cells and activate memory T cells^37,38^. The specificity of alloreactive T cells and the influence of pro-inflammatory conditions on the stimulatory capacity of TEC is displayed by the differing reactivity of CD4^+^ and CD8^+^ T cells. Homeostatic HLA-ABC expression was sufficient to trigger a CD8^+^ T cell response, while CD4^+^ T cells only reacted on inflammatory treated TEC with upregulated HLA-DR molecules. Further, comparing autologous with allogenic stimulation, we could demonstrate that the activation of T cells followed by TEC stimulation was due to allogenic capacity of TEC and not due to unspecific cytokine pre-treatment of TEC. Thus, T cells of healthy volunteers show little to no reaction towards autologous TEC, while allogenic TEC could elicit a measurable reactivity. Taken together, these experiments show that TEC have the ability to induce a specific T cell alloreaction without provoking significant unspecific reactivity.

Knowing that we can specifically monitor TEC-induced donor-reactive T cells, we assessed the sensitivity of our assay in comparison to splenocytes, currently the most commonly used stimulator source. Previously, some authors stated the existence of tissue-specific alloreactivity by HLA-molecules presenting kidney cell specific peptides^39^. Accordingly, splenocytes would only monitor a fraction of the alloreactive T cells as their HLA-molecules do not bind the peptides present in the kidney-allograft. The existence of tissue-specific T cells in the kidney-transplantation setting was already shown more than two decades ago by demonstrating that some clones of graft-infiltrating T cells lyse TEC, but not splenocytes isolated from the corresponding donor^39–45^. In line with these results, we observed a significantly lower reactivity upon stimulation with donor-splenocytes as compared to the donor-derived TEC, despite a higher expression of HLA-molecules on the splenocytes. This underscores the superiority of our TEC-based alloreactivity-assay and suggests that it may reflect donor- and tissue-specific reactivity as well as the intragraft situation more accurately than currently used sources for stimulator cells.

To prove the clinical utility of the established assay, we performed a proof-of-principle study on the correlation of pre-transplant alloreactive T cells measured by the TreaT-assay and the post-transplant GFR as well as early AR. As it has been shown also for the IFNγ-ELISPOT-assay^23–25^, pre-transplant alloreactive CD4^+^ and CD8^+^ T cells inversely correlated with the eGFR at 6 months post transplantation. The prediction of AR with IFNγ-ELISPOT showed differing results in clinical trials^13,23–26,33,34^. Comparing patients with very early AR to patients with a stable graft function in our assay, AR patients showed a higher number of alloreactive CD4^+^ and CD8^+^ T cells and a clear distinction between these two groups can be drawn when the numbers of alloreactive CD161^+^ CD4^+^ T cells and of granzyme B producing CD8^+^ T cells are compared. CD161 is a marker restricted to memory phenotype and a hallmark of TH17 cells and interleukin-17 production^27,46^. Our data are in line with the results by numerous authors demonstrating the involvement of TH17 cells in alloreaction^28^. In addition, Kim *et al*. demonstrated very recently a significant increase of CD161^+^CD4^+^ T cells in patients with antibody-mediated rejection confirming thereby our data on the role of this cell subset in the alloreactivity^47^. The involvement of granzyme B in AR has also been intensely studied and demonstrated before^29^. Taken together, the detection of CD161^+^CD4^+^ and granzyme B in our patients with early AR reported to be relevant in other *ex vivo* and *in vivo* studies provides evidence for the clinical relevance of the data collected.

Our study has some limitations. Similar to other studies performed on peripheral blood cells, our *ex vivo* analyses do not necessarily reflect the intragraft situation. Therefore, in future studies a comparison with the biopsy findings in follow-up would be needed. However, this is challenging due to ethical reasons. Furthermore, the numbers of some alloreactive T cell subsets were relatively low. Detection of very low numbers of antigen-specific T cells in kidney transplant patients has been demonstrated before for virus-specific and vaccine-specific T cells^48–50^. Quality controls for data acquisition and analyses including subtraction of unspecific background and adequate fluorescence-minus-one controls as applied in our studies enable however reliable detection of these cells. On the other hand, analysis of a higher cell number would facilitate identification and in-depth characterization of alloreactive T cells, especially with regard to cytokines or quantification of rare subpopulations. Therefore, drawing higher amounts of blood (e.g. 20 mL) will be advantageous and can be drawn unproblematicly in most cases. Moreover, the number of patients analysed in our proof-of-concept pilot study is insufficient to draw final conclusions on the applicability and prognostic power of our assay and further prospective studies will be required to confirm and extend our data. Additionally, so far we did not perform a direct comparison with similar assays using IFNγ-ELISPOT with donor-splenocytes or HLA-bank cells. Nonetheless, our results are in line with similar assays measuring allograft-reactive T cells in comparable patient cohorts. Further, the time needed for the first results is a critical point in a clinical setting. In order to achieve sufficient numbers of TEC, up to two weeks are necessary, depending on the amount of urine and the sediment quality. However, a combination of the aforementioned sources, such as splenocytes, could bridge this diagnostic window. Another option is down scaling of TEC numbers for stimulation, which is also envisioned for future studies.

Taken together, the TreaT-assay offers a donor-specific measurement of allograft reactive T cells. Compared to previous assays it has the advantages of an unlimited availability and a superior performance. In a pilot study, we were able to obtain encouraging data on the applicability of our assay in patients with early AR and on the prediction of post-transplant GFR. Furthermore, our approach allows deep personalized insights into the biology of alloreactive immune cells and their interaction with donor TEC. Therefore, it might help to guide personalized therapy in the future of kidney transplantation.

## Supplementary information

Supplementary material

## Data Availability

The raw data supporting the conclusions of this manuscript will be made available by the authors, without undue reservation, to any qualified researcher.
