# Supplementary material for: The TreaT-Assay: A Novel Urine-Derived Donor Kidney Cell-Based Assay for Prediction of Kidney Transplantation Outcome
Source: Sci Rep. 2019 Dec 13;9:19037. doi: 10.1038/s41598-019-55442-x (PMC6911059; doi:10.1038/s41598-019-55442-x)
Supplement: Supplementary file 1 — Supplementary material [file 41598_2019_55442_MOESM1_ESM.pdf]

The TreaT-Assay: A Novel Urine-Derived Donor Kidney Cell-Based Assay for Prediction of Kidney Transplantation Outcome

Supplementary Material

Constantin Joachim Thieme [1,2], Benjamin Weist [1], Annemarie Mueskes [1], Toralf Roch [1], Ulrik Stervbo [3], Kamil Rosiewicz [1], Patrizia Wehler [1], Maik Stein [1,4], Peter Nickel [5], Andreas Kurtz [1], Nils Lachmann [6], Mira Choi [5], Michael Schmueck-Henneresse [1,7], Timm Westhoff [3], Petra Reinke [1,4], Nina Babel [1,3,7]*

[1] Berlin Institute of Health Center for Regenerative Therapies (BCRT), Charité – Universitätsmedizin Berlin, Berlin, Germany
[2] Berlin-Brandenburg School for Regenerative Therapies (BSRT), Charité – Universitätsmedizin Berlin, Berlin, Germany
[3] Center for Translational Medicine, Medical Department I, Marien Hospital Herne, University Hospital of the Ruhr-University Bochum, Herne, Germany
[4] Berlin Center for Advanced Therapies (BeCAT), Charité – Universitätsmedzin Berlin, Berlin, Germany
[5] Department of Nephrology and Intensive Internal Care, Charité – Universitätsmedizin Berlin, Berlin, Germany
[6] Zentrum für Transfusionsmedizin und Zelltherapie, Charité – Universitätsmedizin Berlin, Berlin, Germany
[7] Institute for Medical Immunology, Charité – Universitätsmedizin Berlin, Berlin, Germany

*** Correspondence:**
Prof. Dr. med. Nina Babel
nina.babel@charite.de

Patient number	TEC cultivation	HLA Expression on TEC	Establishment of alloreactivity measurement (*number of samples*)	Pre transplant samples - follow up study (** = sample also used in establishment*)	Comparison donor-TEC vs donor-splenocytes (*number of samples*)
#1					
#2					
#3			2		
#4			1		
#5			4	*	
#6			4	*	
#7			2	*	
#8			4	*	
#9			3	*	
#10			1	*	
#11			1	*	
#12			1	*	
#13			2		
#14			3		
#15			2		
#16					
#17					
#18					
#19					4
#20					4
#21					4
#22					3
**Total patients**	**22**	**18**	**13**	**14**	**4**
**Total blood samples**			**30**	**14**	**15**

Supplementary Table S1: Overview of patients and samples analysed in the study

Overview of the patients and their samples used for the corresponding analyses. Green fields indicate that the individual patient has been included into the respective analysis.
